# Evolution and Expansion of the Prokaryote-Like Lipoxygenase Family in the Brown Alga *Saccharina japonica*

**DOI:** 10.3389/fpls.2017.02018

**Published:** 2017-11-28

**Authors:** Linhong Teng, Wentao Han, Xiao Fan, Dong Xu, Xiaowen Zhang, Simon M. Dittami, Naihao Ye

**Affiliations:** ^1^Yellow Sea Fisheries Research Institute, Chinese Academy of Fishery Sciences, Qingdao, China; ^2^Function Laboratory for Marine Fisheries Science and Food Production Processes, Qingdao National Laboratory for Marine Science and Technology, Qingdao, China; ^3^CNRS, UMR 8227, Integrative Biology of Marine Models, Station Biologique de Roscoff, Roscoff, France; ^4^Sorbonne Universités, UPMC Univ Paris 06, UMR 8227, Integrative Biology of Marine Models, Station Biologique de Roscoff, Roscoff, France

**Keywords:** lipoxygenase, brown algae, gene duplication, selection pressure, functional divergence

## Abstract

Lipoxygenase (LOX) plays important roles in fatty acid oxidation and lipid mediator biosynthesis. In this study, we give first insights into brown algal LOX evolution. Whole genome searches revealed four, three, and eleven LOXs in *Ectocarpus siliculosus*, *Cladosiphon okamuranus*, and *Saccharina japonica*, respectively. In phylogenetic analyses, LOXs from brown algae form a robust clade with those from prokaryotes, suggesting an ancestral origin and slow evolution. Brown algal LOXs were divided into two clades, C1 and C2 in a phylogenetic tree. Compared to the two species of Ectocarpales, LOX gene expansion occurred in the kelp *S. japonica* through tandem duplication and segmental duplication. Selection pressure analysis showed that LOX genes in brown algae have undergone strong purifying selection, while the selective constraint in the C2 clade was more relaxed than that in the C1 clade. Furthermore, within each clade, LOXs of *S. japonica* evolved under more relaxed selection constraints than *E. siliculosus* and *C. okamuranus*. Structural modeling showed that unlike LOXs of plants and animals, which contain a β barrel in the N-terminal part of the protein, LOXs in brown algae fold into a single domain. Analysis of previously published transcriptomic data showed that LOXs in *E. siliculosus* are responsive to hyposaline, hypersaline, oxidative, and copper stresses. Moreover, clear divergence of expression patterns was observed among different life stages, as well as between duplicate gene pairs. In *E. siliculosus*, all four LOXs are male-biased in immature gametophytes, and mature gametophytes showed significantly higher LOX mRNA levels than immature gametophytes and sporophytes. In *S. japonica*, however, our RNA-Seq data showed that most LOXs are highly expressed in sporophytes. Even the most recently duplicated gene pairs showed divergent expression patterns, suggesting that functional divergence has likely occurred since LOX genes duplicated, which potentially contributes to the production of various oxylipins in brown algae.

## Introduction

Lipid oxidation is an essential biological process in all living organisms and is subject to both developmental and environmental regulation (Liavonchanka and Feussner, 2006). Lipoxygenases (LOX; EC 1.13.11.12) constitute an important group of enzymes responsible for this kind of lipid metabolism. They are non-haem iron-containing dioxygenases that catalyze the addition of oxygen to polyunsaturated fatty acids (PUFAs) to produce hydroperoxides, which are further converted into a series of biologically active compounds collectively named oxylipins (Fatima et al., 2017). The oxidation of PUFAs via LOXs and the subsequent reactions are collectively called LOX-pathway (Blée, 2002). LOX enzymes are encoded by a multigenic family. They are found in a wide variety of plants, fungi, animals, as well as algae, protozoa, and bacteria (Kalms et al., 2017). The abundant linoleic acid and linolenic acid are major substrates of LOXs in plants, while the principal substrate in animals is arachidonic acid (Porta and Rocha-Sosa, 2001). Derived oxylipins, such as jasmonic acid in plants or prostaglandins and leukotrienes in animals, are important bio-regulators and participate in a wide range of physiological functions, including the regulation of growth, development, and senescence, the mediation of stress responses (Bueno et al., 2001; Hou et al., 2015), or the maintenance of cell homeostasis (Molina and Donaire, 2002; Ackermann et al., 2017).

Lipoxygenases contain a highly conserved region rich in His residues. Five His residues are arranged in the form of His-(X)4-His-(X)4-His-(X)17-His-(X)8-His, which is important for iron atom binding and catalytic activity (Minor et al., 1996). The molecular mass of the LOX proteins identified to date ranges from 75 to 80 kDa in animals, 94–105 kDa in plants and fungi, and 49–75 kDa in bacteria (Feng et al., 2010; Garreta et al., 2013). In both animals and plants, the enzymes possess a small N-terminal domain PLAT/LH2 (homologous to polycystin-1, lipoxygenase, α-toxin, or lipoxygenase) and a major C-terminal domain. With the rapid progress in functional genomics, LOXs have been identified and analyzed in many land plants. At least six LOX genes in *Arabidopsis thaliana* have been identified and expression of AtLOX1 was stimulated by the hormones abscisic acid and methyl jasmonate (MeJA) (Melan et al., 1993). Chen et al. identified 20 LOXs in the poplar genome and showed that many of them have various expression patterns across different tissues and stress treatments (Chen et al., 2015). Chen et al. also investigated the molecular evolution of LOX in modern rosid plants and found that strong purifying selection plays a critical role in LOX evolution (Chen et al., 2016). Lipid peroxidation caused by LOX is of particular importance in fruit ripening and forming volatile flavor aroma, which is a critical aspect of fruit quality. Twelve LOX genes in peach were differentially expressed during fruit ripening under different post-harvest treatments (Guo et al., 2017). In melon and grape berry, members of LOXs also exhibit complex patterns of gene expression in different berry tissues, or during different stages of ripening (Podolyan et al., 2010; Zhang et al., 2014). In pear and apple, an expansion of the LOX family was observed, accompanying a whole genome duplication event that occurred in the common ancestor of the two species. Expression patterns of 18 out of 23 LOXs in pear fruit samples at six different stages after flowering were found to correspond to the changes in the volatile components (Li et al., 2014). These differential expression patterns indicate rapid functional diversification after gene duplication. LOXs are also found in prokaryotes, though in this kingdom of life they are present at much lower frequency (Kalms et al., 2017). The first crystal structure of a prokaryote LOX was obtained in the opportunistic pathogen *Pseudomonas aeruginosa* (Garreta et al., 2013). This LOX was suggested to play a role in the interaction with the host cell membrane. Another study later reported this LOX exhibits a phospholipid oxygenase activity and destroys red blood cells (Banthiya et al., 2015).

LOX-pathway derived oxylipins are also known to be produced in many marine macroalgae. Some of them belong to the prostaglandins and share great similarities with the products of LOXs in animals (Imbs et al., 2001; Potin et al., 2002). The contents of hydroxyl-oxylipins have been quantified in 40 macroalgae, including green, brown, and red algae (Kumari et al., 2014). In the red alga *Rhodymenia pertusa*, four new oxylipins were identified, supporting the existence of a 5*R*-LOX in this species (Jiang et al., 2000). Furthermore, 8-, 9- and 12-LOX pathways were found in other red algae (Gerwick et al., 1999). Brown algae, together with diatoms and oomycetes, are part of an independent lineage, the stramenopiles that evolved from a secondary endosymbiosis event between a protist and an ancestral unicellular red alga. Oxylipins have been reported in several bloom-forming diatoms (Wichard et al., 2005). Polyunsaturated aldehydes have, for instance, been detected in the marine diatom *Thalassiosira rotula*, where LOX-mediated fatty acid transformation might function as chemical defense by reducing the hatching success from eggs of copepods (Pohnert, 2002), whereas the synthesis of oxylipins in *T. rotula* relies on an unusual heterolytic mechanism involving a multifunctional LOX to perform both hydroperoxidation and cleavage of hydroperoxide (Barofsky and Pohnert, 2007). Another example is the pennate diatom *Pseudo-nitzschia delicatissima*, which produces three different species of oxylipins via the 15-LOX pathway (d’Ippolito et al., 2009). Kousaka et al. (2003) also found a multitude of oxylipin metabolites in the brown alga *Eisenia bicyclis*.

Oxylipin studies in macroalgae have been mainly focused on their isolation, structural characterization, and biological properties. The past decade has seen a few studies on deciphering the roles of macroalgal oxylipins in defense and immunity. Brown algae contain high amounts of both C18 and C20 fatty acids (Hofmann and Eichenberger, 1997; Küpper et al., 2009). In the brown alga *Laminaria digitata*, arachidonic acid, linolenic acid, and MeJA were strong triggers of an oxidative burst, and brought about changes in fatty acid and oxylipin profiles (Küpper et al., 2009), which suggests that the lipid oxidation pathway plays a major role as a defense mechanism in brown algae. Oxylipins derived from oxidation of polyunsaturated fatty acids (PUFAs) in brown algae were responsive to external stimuli or mezo-grazers (Pohnert and Boland, 2002). Prostaglandin A2 has been reported to trigger an oxidative burst in *Laminaria* sp. and may constitute a novel defense inducer in brown algae (Zambounis et al., 2012). Copper stress in the brown alga *L. digitata* induced the production of as many as 23 oxylipins, and treatment with the inhibitors of LOX enzymes efficiently reduced oxylipin production (Ritter et al., 2008). Ritter et al. (2014) also reported the detection of diverse oxylipins produced via the LOX pathways in the copper-stressed *Ectocarpus siliculosus*.

Although LOX products have been described from Phaeophyceae, to the best of our knowledge, there has been no comprehensive study of LOX genes in brown algae available up to now, and their phylogenetic relationships with LOXs of other phyla remain poorly understood. Brown macroalgae are the dominant vegetation in many benthic marine habitats with high ecological and economic significance (Xu et al., 2017). Particularly, *Saccharina japonica* is one of the most economically important seaweed in marine aquaculture. The completion of genome sequencing projects for brown algae *Ectocarpus* (Cock et al., 2010), *S. japonica* (Ye et al., 2015) and *Cladosiphon okamuranus* (Nishitsuji et al., 2016) greatly facilitate exhaustive inventories of gene families and cross phyla comparisons. Furthermore, comparative genomics between closely related species provides a way to understand evolution and function of genes in multigene families and their roles in adaptation to ecological niches (Good et al., 2014). In this study, we characterized the brown algal LOX family and attempted to determine their expansion mechanism in *S. japonica*. Such knowledge will increase our understanding of the roles of LOXs and oxylipins in brown algal development as well as facilitate the utilization of these algal enzymes as tools in synthetic chemistry for the production of oxylipins.

## Materials and Methods

### Database Search and LOX Sequence Retrieval

Protein and CDS sequences of *E. siliculosus* V2016 version were downloaded from the website http://bioinformatics.psb.ugent.be/orcae/overview/Ectsi. Those of *S. japonica* were downloaded from NCBI under the accession code JXRI00000000. Sequences of *C. okamuranus* were downloaded from http://marinegenomics.oist.jp/algae/. Other genomes were downloaded from the following sites: green plants, Phytozome V12.0 https://phytozome.jgi.doe.gov/pz/portal.html. *Emiliania huxleyi*, oomycetes, http://genome.jgi.doe.gov/pages/tree-of-life.jsf. Other genes were downloaded at NCBI https://www.ncbi.nlm.nih.gov/. The LOX domain PF00305 was downloaded from the Pfam website http://pfam.xfam.org/ (Finn et al., 2015). HMMER was used to search for this domain in the predicted proteins of each species. All of the obtained LOXs were further manually examined using the NCBI online BLAST tool https://blast.ncbi.nlm.nih.gov/Blast.cgi to confirm the genes’ annotation. The online InterProScan program http://www.ebi.ac.uk/interpro/search/sequence-search was used to confirm the presence of LOX domain in each sequence.

### Phylogenetic Analysis

Sequence alignments were performed on both the full-length proteins and the conserved PF00305 domain using MUSCLE embedded in MEGA 7.0 (Kumar et al., 2016). The maximum likelihood (ML) and Neighbor-joining (NJ) phylogenetic trees were constructed with best substitution models and rate parameter WAG + G + I found by MEGA 7.0. Bootstrap with 1000 bootstrap replicates was performed to obtain the confidence support level. Bayesian inference of phylogeny was carried out using BEAST2 (Bouckaert et al., 2014). Analyses were run for 100 million generations, with sampling every 10000th generation using the same substitution model as above and Yule Model as a prior. The first 10000 samples were discarded as burn-in.

### Sequence Structure Analysis

Intron and exon information of the three brown algal LOX genes was obtained from their GFF files and was used for the graphic display using the Gene Structure Display Server of Peking University (Hu et al., 2015). The protein transmembrane helices were predicted by CCTOP^1^ (Dobson et al., 2015). Structural motifs were searched for using the MEME program http://meme-suite.org/ (Bailey et al., 2009). The motif number was set to 15. The most conserved motif of LOXs was displayed on the WebLogo website http://weblogo.berkeley.edu/logo.cgi (Crooks et al., 2004). Signal peptides were predicted using the website http://www.cbs.dtu.dk/services/SignalP/ (Petersen et al., 2011). Subcellular protein localization was predicted using ASAFind (Gruber et al., 2015). Mass weight (MW) and isoelectric point (p*I*) was calculated using the website http://www.expasy.org/.

### Molecular Evolution Analysis

In total, 18 protein sequences of LOXs in three brown algae *Ectocarpus*, *S. japonica*, *C. okamuranus* were aligned using MUSCLE. A ML tree was constructed with MEGA 7.0 as described above. Brown algae LOXs were divided into two clades C1 and C2. In each clade, the branches were divided again into Ectocarpales clades (C-E) and Laminariales clades (C-S). To evaluate variation in selective pressures between different clades (C1-E *vs.* C1-S; C2-E *vs.* C2-S), a branch model in PAML v4.8 (Yang, 2007) was used to estimate the ω (d*N*/d*S*) ratio under two assumptions: a one ratio model (*M* = 0) that assumes the same ω in C1 and C2 clade, respectively; a two-ratio model (*M* = 2) that assumes that Ectocarpales clade (C-E) and *Saccharina* clade (C-S) have different ω ratios. To test which of the models best fit the data, likelihood ratio tests (LRTs) were performed by comparing twice the difference in log-likelihood values using a χ2 distribution with one degree of freedom. We also ran site models (*M* = 0, NSsites = M0, M3, M7, M8) in Codeml to test if there were sites of positive selection in brown algal LOXs. LRTs were performed (M0 *vs.* M3, M7 *vs.* M8) to verify which model fit the data using a χ2 distribution with two degrees of freedom. To further explore the evolutionary dynamics of expanded LOXs in *S. japonica*, pairwise d*N*, d*S* and d*N*/d*S* values between duplicated pairs of *S. japonica* LOXs were calculated using the online PAL2NAL program http://www.bork.embl.de/pal2nal/index.cgi (Suyama et al., 2006).

### Expression of LOX Genes in *E. siliculosus* and *S. japonica*

Previous microarray data of the *E. siliculosus* transcriptome (Dittami et al., 2009; Ritter et al., 2014) was used to explore the expression levels of LOXs in response to abiotic stressors, notably hyposaline stress, hypersaline stress, oxidative stress, and Cu stress. The expression level was determined by averaging the expression values (previously quantile normalized by Roche NimbleGen, Madison, WI, United States) of four replicates for each experimental condition. Furthermore, expression levels of LOXs were examined in different life cycle stages. In *E. siliculosus* we compared previously published RNA-Seq data of parthenosporphytes, male gametophytes, and female gametophytes (Lipinska et al., 2015). Expression levels of *S. japonica* LOXs were examined in sporophytes, male gametophytes, and female gametophytes. Their transcriptomes were sequenced by our group using an Illumina HiSeq 2500, with three biological replicates in each life stage. Gene expression levels were estimated by FPKM (Fragments Per Kilobase of transcript per Million fragments mapped). Differential expression analysis among life stages was performed using the DESeq (Love et al., 2014) R package using a model based on the negative binominal distribution. The resulting *p*-values were adjusted using the Benjamini and Hochberg’s approach for controlling the false discovery rate (Benjamini and Hochberg, 1995). Genes with an adjusted *p*-value < 0.05 found by DESeq were assigned as differentially expressed. The raw sequence data was deposited at NCBI database. The SRA accession numbers are SRR5860560, SRR5860561, SRR5860562, SRR5860563, SRR5860564, SRR5860565, SRR5860566, SRR5860567, and SRR5860568.

### Structure Modeling

To predict the 3D structures of *S. japonica* LOX proteins, the X-ray structure of a *P. aeruginosa* LOX protein (PDB accession numbers 4g33.1, 5lc8.1, 4rpe.1) was used as template, as it has the highest coverage and similarity with *S. japonica* LOXs. Each LOX was aligned with the template and the structure was automatically built using SWISS-MODEL https://www.swissmodel.expasy.org/ (Biasini et al., 2014). Secondary structure was displayed using the Espript web server http://espript.ibcp.fr/ESPript/cgi-bin/ESPript.cgi (Gouet et al., 2003).

## Results

### Phylogenetic Analysis and Structures of the LOX Gene Family in Brown Algae

**FIGURE 1:**
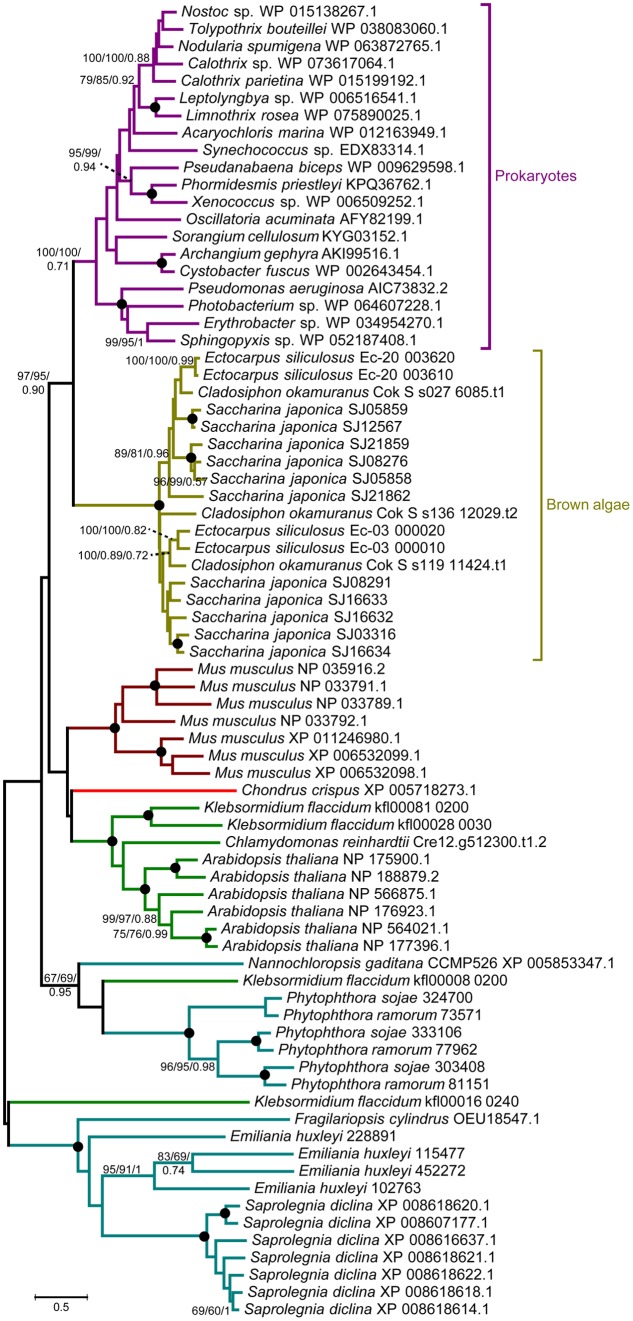
Unrooted ML tree of 76 members of the LOX family from prokaryotes and 14 eukaryote species. Support values correspond to bootstrap values (1000 replicates) for ML and NJ trees and Bayesian posterior probabilities in this order; they are shown only for nodes with ≥50% support with all three methods. Nodes with 100% in all methods are shown by a solid dot. The tree was generated using MEGA 7.0 and WAG + G + I model for ML and NJ analyses and BEAST2 for Bayesian methods.

A comprehensive genome-wide search identified 12, 4, and 3 LOX genes in genomes of the *S. japonica, E. siliculosus*, and *C. okamuranus*, respectively. The number of LOX genes in *S. japonica* is approximately three-fold that in *E. siliculosus* and *C. okamuranus*. The accuracy of these gene models was manually checked by comparison with publicly available RNA-Seq data. We found a large number of reads spanning the SJ21862 and SJ21863, suggesting they correspond to a single transcript encoding a 518 residue protein. Furthermore, SJ16633 and SJ16634 were truncated at the N-terminal end and manually extended increasing their length by 25 and 91 residues, respectively. Additionally, SJ08291 and SJ05859 have the shortest protein length, and no expression data available, suggesting they are probably pseudogenes. Detailed information about the LOX family in brown algae is listed in **Table 1**. The length of these proteins ranged from 346 to 747 amino acid residues. The molecular masses ranged from 39 to 82 kDa, with SJ08291 and Ec-03_000010 being the smallest and largest, respectively. The p*I* values of these proteins are similar, ranging from 4.65 to 5.79. In *S. japonica*, *E. siliculosus*, and *C. okamuranus*, three, one, and two LOXs were predicted to be located in the chloroplast, respectively. In order to perform phylogenetic analysis, we also searched for the LOX genes in other organisms, including oomycetes, *Phaeodactylum tricornutum*, *Thalassiosira pseudonana*, *Fragilariopsis cylindrus*, *Nannochloropsis gaditana*, and *Emiliania huxleyi*, all of which, together with brown algae belong to the Stramenopiles and Haptophytes. Besides, LOX in prokaryotes, model plants and animal were included. Sequences corresponding to the LOX domain (PF00305) were aligned using MUSCLE imbedded in MEGA 7.0. Then ML and NJ trees were constructed using MEGA 7.0, and a Bayesian tree was constructed using BEAST 2. The three trees exhibited nearly the same topology. As shown in **Figure 1**, brown algal LOXs formed one robust clade, with 100% bootstrap support. It is interesting that LOXs of brown algae are most closely related to those of prokaryotes with high bootstrap support but not with other Stramenopiles, plants, or animals. From this tree topology, we propose that LOXs in both brown algae and prokaryotes, as well as LOXs in plant/animal or haptophytes/oomycetes originate from several different ancestral LOXs, and that each lineage retained different copies.

**Table 1:** Detailed information about the LOX gene family in brown algae.

ID	Length	MW	p*I*	TH	ASAFind	
	(a.a.)	(Da)			prediction	
					
					SP	Chloroplast
SJ03316	671	73402.1	5.10	0	Y	–
SJ05859	405	43877.2	5.51	1	–	–
SJ05858	669	74666	4.80	0	–	–
SJ08276	666	74130.5	4.77	0	Y	Y
SJ08291	346	38987.2	5.30	0	–	–
SJ12567	654	71750.7	5.79	0	–	–
SJ16633	558	62434.2	5.01	0	–	–
SJ16634	737	80816	5.31	0	Y	–
SJ16632	673	74173.2	5.14	0	Y	Y
SJ21862	518	57993.4	4.65	0	–	–
SJ21859	666	74062.6	4.87	0	Y	Y
Ec-03_000010	747	82237.2	5.22	0	–	–
Ec-03_000020	604	67597	5.18	0	–	–
Ec-20_003610	730	81848.8	4.84	0	–	–
Ec-20_003620	652	73388.1	4.80	0	Y	Y
Cok_S_s027_6085.t1	672	74642.6	4.95	0	Y	Y
Cok_S_s119_11424.t1	657	72334	4.86	0	Y	–
Cok_S_s136_12029.t2	496	54558.5	5.41	0	Y	Y


*TH, transmembrane helices; SP, Signal peptide.*

**FIGURE 2:**
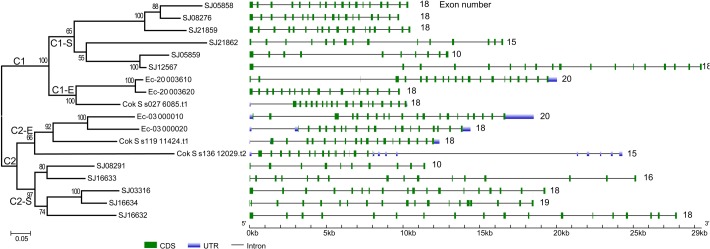
Phylogenetic relationship and gene structure of brown algal LOX genes. The phylogenetic tree was constructed by the ML method with 1000 bootstrap replicates. The two LOX clades were designated as C1 and C2, in which the subclades were designated as C1-S, C1-E, C2-S, C2-E, respectively. Exons and introns are represented by green boxes and black lines, respectively.

**FIGURE 3:**
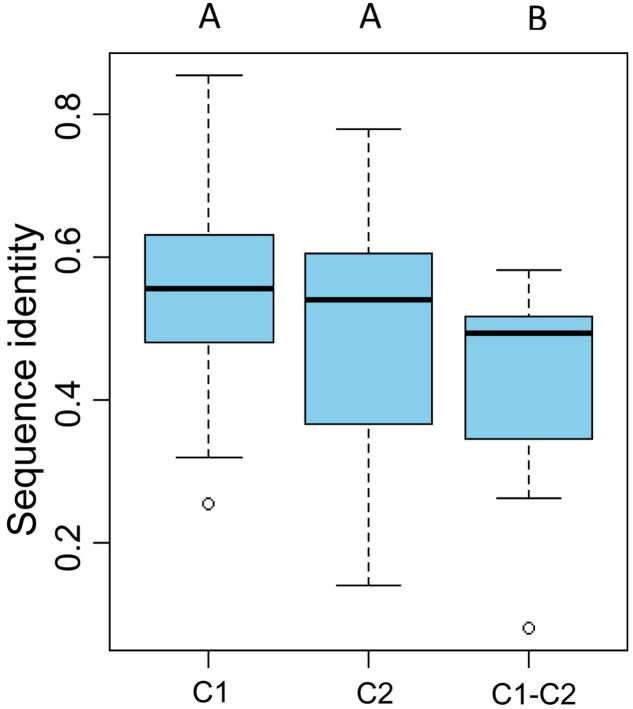
Pairwise sequence identity of full-length LOX proteins. C1 and C2 represent pairwise sequence identities of clade 1 and clade 2 proteins, respectively. C1–C2 represent pairwise sequence identities between the C1 and C2 clades. The boxplot shows the median (black line), interquartile range (box), and maximum and minimum values (whiskers) for each data set. Circles are outliers. Characters above the figure indicate significant differences between groups at *p* = 0.01, Mann–Whitney test.

To gain further insight into the diversity of brown algal LOXs, we constructed a separate phylogenetic tree using only the brown algal LOX protein sequences. The tree showed that the 18 LOXs were divided into two distinct clades (C1 and C2, **Figure 2**). C1 and C2 contained 9 genes each. The C1 clade was further divided into two subclades (C1-S and C1-E). Similarly, the C2 clade also contained two subclades (C2-S and C2-E), in which the ‘S’ and ‘E’ denote *Saccharina* and Ectocarpales, respectively. Subclades C1-E and C2-E contained LOXs from *E. siliculosus* and *C. okamuranus*, whereas subclasses C1-S and C2-S contained only LOXs from *S. japonica*. Striking gene structure conservation was found within 18 LOXs of brown algae in terms of either intron numbers or exon length. A total of 15 out of 18 genes contain from 15 to 20 introns, of which ten genes contain 17 introns with exons of similar length. Fewest exons were seen in SJ05859 and SJ08291, with only ten exons, which further supported that they are possibly pseudogenes. Intron length in *S. japonica* varies from 39 bp in SJ16634 to 9,722 bp in SJ12567 whereas that in *E. siliculosus* varies from 159 bp in Ec-03_000010 to 6,520 bp in Ec-20_003610. Pairwise comparison of the 18 LOX full-length protein sequences revealed some features (**Figure 3**). C1 clade proteins showed 25.4–85.5% pairwise sequence identity. C2 clade showed 14.1–78.1% pairwise sequence identity. However, the protein sequences between the two LOX clades were significantly more different (Mann–Whitney test, *p* < 0.01) and the pairwise sequence identity was 8.2–58.2% (Supplementary Table S1).

**FIGURE 4:**
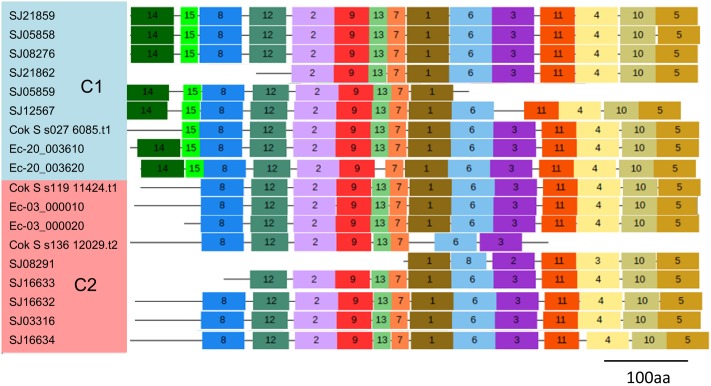
Schematic representation of the conserved motifs in brown algal LOX proteins. Each colored box represents a motif in the protein, with the motif number indicated in the middle of the box. The length of the protein and motif can be estimated using the scale at the bottom.

**FIGURE 5:**
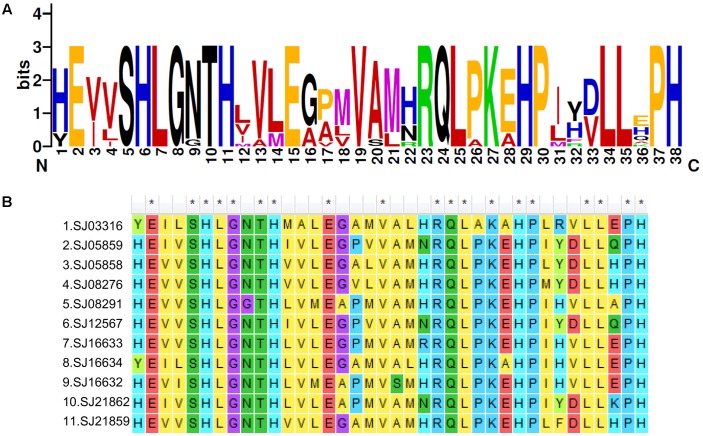
A 38-residue motif among *S. japonica* LOX sequences. **(A)** The logo was created with 11 *S. japonica* LOX sequences. The overall height of each stack indicates the sequence conservation at that position and the height of each residue letter indicates the relative frequency of the corresponding amino acid residue. **(B)** Sequence alignments of 38-residue motifs in *S. japonica* LOX members.

To explore the detailed motif composition of the brown algal LOX family, we predicted putative motifs using the online tool MEME (Bailey et al., 2009). In total, 15 motifs were identified (**Figure 4** and Supplementary Table S2). The presence of conserved motifs in LOX proteins provides additional support for the results of the phylogenetic analysis. Compared with the C2 clade, two distinct motifs occurred only in the C1 clade, that is, motifs 14 and 15 in the N-terminal region of the protein. However, whether these motifs confer a specific function to members of the C1 clade remains to be surveyed. Motifs 1 and 6 contain the representative 38 amino acid residues with the structure His-(X)4-His-(X)4-His-(X)17-His-(X)8-His. In the protein sequence alignment of *S. japonica*, all of the nine sequences have the conserved motif in this His rich region, except two sequences (SJ03316 and SJ16634) where the first His is replaced by Tyr (**Figure 5**). Motifs 4 and 5 contain an Asn and Ile residue respectively, which are essential for the binding of iron. Notably, some LOXs lack critical motifs. For instance, SJ05859 lacks the six motifs in C-terminal whereas SJ08291 lacks the eight motifs in N-terminal. One gene in *C. okamuranus* lacks motif 1 and four motifs in C-terminal.

### Molecular Evolution

The ratio of non-synonymous (d*N*) to synonymous (d*S*) nucleotide substitutions ω (d*N*/d*S*) provides information about the evolutionary forces operating on a gene or gene region (Yang et al., 1998). ω < 1 suggests negative selection and the removal of adverse mutation. ω = 1 and ω > 1 suggest neutral selection and positive selection, respectively. The LOX family of brown algae was divided into two clades (C1 and C2), and they were further divided into subclades (C1-S and C1-E, C2-S and C2-E). To infer the selection pressure within each clade, we estimated ω values with branch models using the CODEML program in the PAML software package (**Table 2**) (see Materials and Methods). The log-likelihood values under the one ratio model were lnL = -8197.2974 and -7447.3493, with estimates of ω = 0.10530 and 0.1262 for the C1 and C2 clades LOX, respectively. Under the two-ratio model, the log-likelihood values were lnL = -8193.6592 and -7441.3471 for the C1 and C2 clade LOX genes, respectively. Likelihood ratio tests (LRTs) of the two assumptions indicated that the two-ratio model was significantly more likely (*p* < 0.01) for both the C1 and C2 LOX clades, suggesting that selective pressure varied among LOX subclades (C1-S *vs.* C1-E, C2-S *vs.* C2-E). LOX genes in *S. japonica* have experienced more relaxed purifying selection than those in *E. siliculosus* and *C. okamuranus*. All of the estimated ω values were <1, suggesting that the two LOX clades have been under purifying selection but that the selection constraint on the C2 clade was more relaxed than that on the C1 clade, which is consistent with the lower sequence identity in the C2 clade. However, the ratio averaged over all sites is unlikely to exceed 1, because positive selection is not likely to affect all codon sites of a gene (Yang, 2007). Site model allows the ω ratio to vary among sites over all branches (Roux et al., 2014). Therefore, we employed site models (M0 *vs.* M3, M7 *vs.* M8) to test whether episodic positive selection had affected specific amino acid sites in each gene. M0 assumes one same ratio among all sites whereas M3 assumes discrete ratios among sites. Under the M7 model, ω follows a beta distribution between 0 and 1, while no ω > 1 site allowed. The M8 model allows an additional site class with ω > 1 (Yang, 2007). LRT was performed to test which model fits the data best. The site model analysis between M0 and M3 indicated that selection pressure varied among sites both for C1 and C2 clades (*p* < 0.001). However, there is no significant difference between the lnL values of M7 and M8, indicating that no site was positively selected, which further supported the results from branch model.

**Table 2:** Summary statistics for detection of selection using branch and site models.

Clade	Model	Estimates of Parameters	-lnL	χ^2^	*P*
	**Branch model**				
C1	One ratio	ω = 0.10530	8197.2974		
	Two ratios	ω0 = 0.11443 for clade C1-S	8193.6592	7.2764	0.007
		ω1 = 0.04887 for clade C1-E			
C2	One ratio	ω = 0.1262	7447.3493		
	Two ratios	ω0 = 0.13739 for clade C2-S	7441.3471	12.0044	<0.001
		ω1 = 0.05425 for clade C2-E			
	**Site model**				
C1	M0	ω = 0.10530	8197.2974	319.1584	<0.001
	M3	p0 = 0.36492, p1 = 0.51998, p2 = 0.11510	8037.7182		
		ω0 = 0.00000, ω1 = 0.14519, ω2 = 0.58148			
	M7	p = 0.45198, q = 2.69774	8041.8773		
	M8	p0 = 0.99414, p = 0.46841, q = 2.90150,	8040.1616	3.4314	
		(p1 = 0.00586), ω = 4.62816			
C2	M0	ω = 0.1262	7447.3493	228.6478	<0.001
	M3	p0 = 0.21404, p1 = 0.44069, p2 = 0.34527	7333.0254		
		ω0 = 0.03836, ω1 = 0.03836, ω2 = 0.36779			
	M7	p = 0.50236, q = 2.70199	7334.6504	0.4212	
	M8	p0 = 0.99711, p = 0.51412, q = 2.82023,	7334.4398		
		(p1 = 0.00289), ω = 2.62022			


### Gene Duplications in the LOX Gene Family of Brown Algae

**FIGURE 6:**
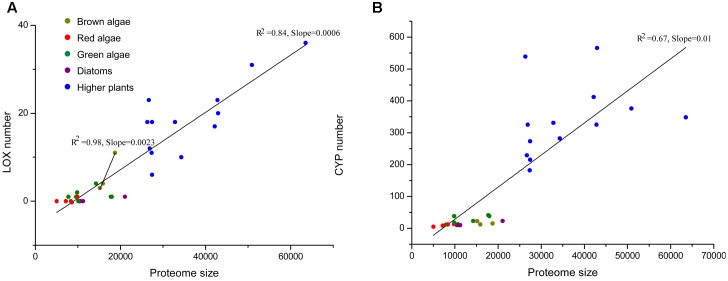
**(A)** The total number of LOXs relative to the total proteome size in a selection species. The following species were included: higher plants – *Arabidopsis thaliana*, *Prunus persica*, *Populus trichocarpa*, *Brachypodium distachyon*, *Medicago truncatula*, *Oryza sativa*, *Vitis vinifera*, *Carica papaya*, *Cucumis sativus*, *Pyrus bretschneideri*, *Malus domestica*, *Fragaria vesca*, *Cucumis melo*; red algae – *Chondrus crispus*, *Galdieria sulphuraria*, *Cyanidioschyzon merolae*, *Porphyridium purpureum*; diatoms – *Thalassiosira pseudonana*, *Phaeodactylum tricornutum*, *Fragilariopsis cylindrus*; green algae – *Chlamydomonas reinhardtii*, *Chlorella variabilis*, *Coccomyxa subellipsoidea*, *Gonium pectoral*, *Micromonas pusilla*, *Micromonas* sp. RCC29, *Ostreococcus lucimarinus*, *Volvox carteri*. Data for higher plants were taken from Feng et al. (2010); Podolyan et al. (2010); Liu et al. (2011); Li et al. (2014); Zhang et al. (2014); Chen et al. (2015, 2016); Guo et al. (2017). **(B)** Another family of metal-binding proteins, the cytochromes P450 (CYP) family is displayed as a control set.

In order to illustrate the expansion of LOXs in *S. japonica*, we compared the LOX number across different organisms. Proteome size-dependent expansion of metal-binding proteins was observed across all domains of life (Dupont et al., 2010). Thus, the relationship between LOX number and proteome size was taken into consideration (**Figure 6**). LOXs are rare among most sequenced algae. The green alga *Volvox carteri* has four members, while other green algae, red algae, and diatoms have maximally one LOX. The two brown algae *E. siliculosus* and *C. okamuranus* have four and three LOXs, respectively. Notably, *S. japonica*, which possesses 11 LOXs, has expanded its LOX family compared with other algae. Furthermore, this expansion goes beyond the mere effect of proteome size, as indicated by the different slopes for brown algae and other algae in the linear regressions in **Figure 6A**. Moreover, a similar effect was not observed for other metal-binding protein families such as the cytochromes P450 (CYP) family (**Figure 6B**).

**FIGURE 7:**
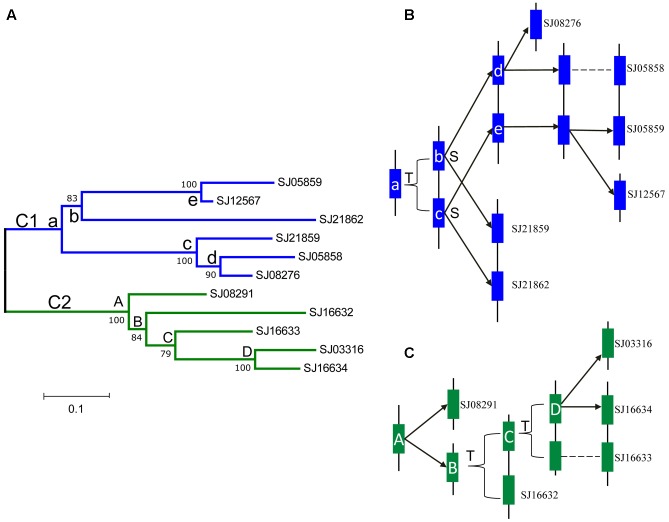
Phylogenetic relationships and hypothetical evolutionary histories of the *S. japonica* LOX genes in Class 1 and 2. **(A)** Phylogenetic relationships were reconstructed using MEGA7.0. **(B)** The duplication histories of Class 1. **(C)** The duplication histories of Class 2. The letters T and S indicate putative tandem duplication and segmental duplication events, respectively. The letters in the tree and blue, green boxes represent ancestral LOX genes.

According to the physical locations of the LOXs on chromosomes or scaffolds, gene duplication events were constructed. In *E. siliculosus*, the four LOX genes were assigned to two chromosomes. Within each chromosome, tandem duplication occurred once. However, LOX genes in *S. japonica* have undergone more rounds of duplication and formed a more expanded LOX gene family compared to *E. siliculosus*. Tandem duplication plays a major role in the *S. japonica* LOX expansion history. Three tandem clusters account for 7 out of 11 LOX genes, including SJ05858/SJ05859, SJ21859/SJ21862, and SJ16632/SJ16633/SJ16634. Other genes likely came from segmental duplication after a series of tandem duplications. The d*N*, d*S* and d*N*/d*S* ratio of these putative paralogous gene pairs were calculated to investigate the divergence fate after duplication (**Table 3**). All d*N*/d*S* ratios were <0.5, suggesting strong purifying selection, which is consistent with the branch/site model results. The largest ratio was observed in the gene pair SJ05859/SJ12567 with the value of 0.4393, indicating the relatively relaxed selection pressure between them. Duplicate paralogs diverge from the ancestral state and accumulate synonymous mutations at similar rates over time (Holland et al., 2017), so the d*S* value can be used to measure the time since gene divergence. The lowest d*S* (0.0520) was observed between Ec-20_003610/ Ec-20_003620, suggesting that they were created most recently, whereas the highest d*S* (1.6936) was found between SJ21859/SJ21862, suggesting an ancient duplication, likely derived from the first round of ancestral tandem duplications. By reconciliation of the physical position of genes, gene tree, and the d*S* values, we attempted to reconstruct the most parsimonious history of *S. japonica* LOX gene duplication (**Figure 7**). For the C1 clade, it seems that one tandem duplication created two ancestral genes (b and c), after which other events, likely segmental duplications, occurred to form two gene pairs; one pair is SJ21859 and SJ21862, the other pair is ancestral d and e. After that, two duplication events occurred sequentially, to create two more pairs of LOXs. On the other hand, in the C2 clade, ancestral gene A duplicated to form SJ08291 and ancestral B, followed by two rounds of tandem duplications to create SJ16632 and ancestral C, D, which further produced SJ16633, SJ16634, and SJ03316.

**Table 3:** Divergence between paralogous LOX gene pairs in brown algae.

No.	Gene 1	Gene 2	d*N*	d*S*	d*N*/d*S*	Gene expression (mean FPKM for Sj, RPKM for Es)	
						
						Gene 1	Gene 2
1 T	C1: SJ05859	SJ05858	0.2320	1.5570	0.1490	S:0.08	S:130.54, M:0.40, F:1.27
2 T	C1:SJ21859	SJ21862	0.2456	1.6936	0.1450	S:102.85	S:0.20
3 T	C2:SJ16633	SJ16632	0.1450	1.0390	0.1396	S:201.99; M:1.56, F:11.98	S:106.44, M:4.20, F:1.87
4 T	C2:SJ16633	SJ16634	0.1197	0.8038	0.1489	S:201.99; M:1.56, F:11.98	S:4.09
5 T	C2:SJ16632	SJ16634	0.2025	1.1486	0.1763	S:106.44, M:4.20, F:1.87	S:4.09
6 T	C2:Ec-03_000010	Ec-03_000020	0.1149	0.7285	0.1577	S:140.9; M:241.7-310.9; F:49.1-584.6	S:20.5; M:84.8-127.4; F:31.7-188.7
7 T	C1:Ec-20_003610	Ec-20_003620	0.0200	0.0520	0.3843	S:0.2; M:3.7-2.9; F:0.9-6.1	S:12.0; M:648.7-490.5; F:219.5-630.7
8 O	C1:SJ08276	SJ05858	0.0722	0.4778	0.1510	S:4.72	S:130.54, M:0.40, F:1.27
9 O	C1:SJ05859	SJ12567	0.0479	0.1089	0.4393	S:0.08	S:4.25, M:30.22, F:1.26
10 O	C2:SJ03316	SJ16634	0.0789	0.3479	0.2269	S:0.06	S:4.09


*T, tandem duplication; O, other duplication; S, expressed in sporophytes; M, expressed in male gametophytes; F, expressed in female gametophytes; In E. siliculosus, the numbers connected by hyphen denote the expression values in immature and mature gametophytes, respectively.*

### Expression of LOX Genes in Brown Algae

**FIGURE 8:**
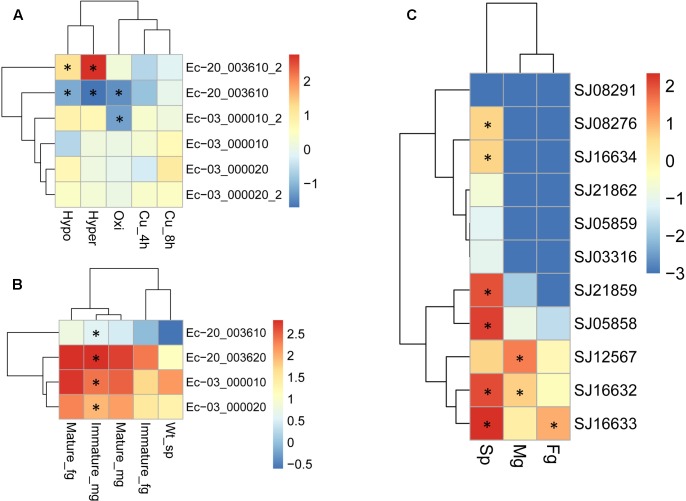
Expression profiles of LOX genes in *E. siliculosus* **(A,B)** and *S. japonica* **(C)** respectively. **(A)** Log2-transformed fold changes of expression levels compared to the control. Black star indicates the log2 values of fold changes (treatments/control) > 1.0 or < -1.0. **(B)** Log10-transformed RPKM values. Black star indicates the sex-biased expression compared between male and female gametophytes (fold changes > 2, adjusted *p*-value < 0.05). **(C)** Log10-transformed FPKM values. Black star indicates sex-biased or sporophyte-biased expression (fold changes > 2, adjusted *p*-value < 0.05). sp, sporophytes; fg, female gametophytes; mg, male gametophytes.

Duplicated genes may evolve into different fates, which can be inferred by the divergence of their expression patterns. The expression patterns of LOX gene family members were examined using the publically available microarray data of *E. siliculosus* and RNA-sequencing data of *E. siliculosus* and *S. japonica*. Three of the four LOX genes in *E. siliculosus* are present in the microarray data, and all the three genes have two contigs/singletons. Hierarchical clustering showed that expression levels under Cu stress were clustered with oxidative stress (**Figure 8A**), which is consistent with the meta-analysis in Ritter et al. (2014). One gene (Ec-20_003610) was significantly influenced by hyposaline, hypersaline, and oxidative stress (log2-fold change > 1 compared to control). The other two genes were up-regulated by hyposaline, hypersaline, and Cu stress, though the log2-fold change did not reach one. This result indicates that LOX genes in *E. siliculosus* are responsive to these stress conditions, and may explain the observed accumulation of oxylipins under Cu stress (Ritter et al., 2014). Expression divergence was also observed in different life stages. In *E. siliculosus*, mature male and female gametophytes have significantly higher LOX mRNA levels than sporophytes or immature gametophytes. Notably, all of the four LOXs were male-biased in immature gametophytes (fold change > 2, adjusted *p*-value < 0.1; **Figure 8B**), suggesting their potential roles in sexual dimorphism. For the LOX members in *S. japonica*, more variation in expression patterns was found in different life stages (**Figure 8C** and **Table 3**). Three genes were sex-biased, including one female-biased (SJ16633) and two male-biased (SJ16632 and SJ12567). Two genes in the C1 clade (SJ05858, SJ21859) and two genes in the C2 clade (SJ16632, SJ16633) were ranked as the most highly expressed in sporophytes. However, expression levels of other genes were relatively low, especially in gametophytes. Two genes (SJ21862, SJ03316) were exclusively expressed in sporophytes, whereas no expression was detected in gametophytes, suggesting that they are likely only functional in sporophytes. The other genes also exhibited higher expression levels in sporophytes than in gametophytes, except for one gene (SJ12567), which showed the highest expression in male gametophytes. Another gene (SJ08291) was not expressed in any life stage, and SJ05859 had very a low FPKM of only 0.08. These two genes are likely to be pseudogenes, considering their gene structure and expression level. Notably, the two pairs of LOX genes with lowest expression levels (SJ05859 *vs.* SJ12567, SJ03316 *vs.* SJ16634) have higher d*N*/d*S* values than other LOX gene pairs (**Table 3**). Likewise, in *E. siliculosus*, the lowest overall expression level (Ec-20_003610) and great expression difference was observed in the gene pair with the highest dN/dS value (0.3843 compared to 0.1577 in the other pair of genes). The expression patterns of LOX suggested that functional diversification has occurred between the duplicated LOX gene pairs.

### Structure Modeling

**FIGURE 9:**
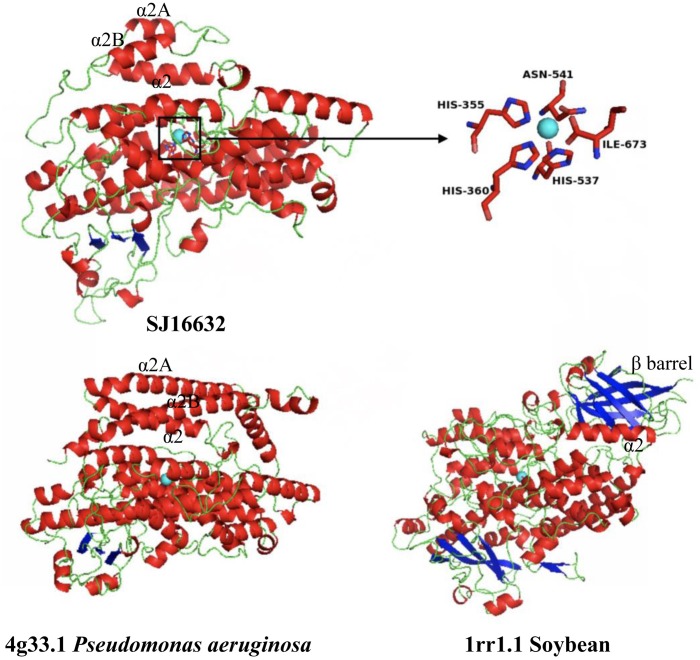
Modeled 3D structure of the *S. japonica* LOX protein (SJ16632) and the 3D structure of *P. aeruginosa* and soybean. Blue sphere: non-heme iron; inset: the predicted active site for binding iron.

Three-dimensional protein structure modeling across all eleven LOXs in *S. japonica* revealed that, in general, these proteins are best modeled by a 15-LOX in the bacterium *P. aeruginosa* (coverage 79–89%, identity 27.83–41.51%), which further supports the close relationships between them. The simulated structures of *S. japonica* LOX showed remarkable differences to plant or animal LOXs. For most plant and animal LOXs, a two-domain structure has been identified, but LOXs in brown algae fold into a single domain. The helical lid structure in the *P. aeruginosa* LOX was suggested to be the functional equivalent of the N-terminal β-barrel in eukaryotic LOXs (Garreta et al., 2013). In LOXs of *S. japonica*, the N-terminal part seems to also contain the helical lid structure (α2A, α2B), though having shorter helices than the *P. aeruginosa* LOX (**Figure 9** and Supplementary Figure S1). The X-ray structure of 4g33.1 showed that five sites, His377, His382, His555, Asn559, and Ile685 are essential for binding of iron (Garreta et al., 2013; Kalms et al., 2017). Sequence comparison showed that the five sites were conserved in all the *S. japonica* LOXs, except for protein SJ05859 due to the short sequence length. The predicted 3D structure of SJ16632 showed that the five sites could interact with the iron. The simulated structures of six LOXs in the C1 clade and five LOXs in the C2 clade were superimposed to evaluate the goodness of fit of the overall topologies (Supplementary Figure S2). The result shows that the structure of the α-helix is conserved across all members, whereas structures in the loop regions are variable and less conserved, which might be due to relaxed functional constraints.

## Discussion

Lipoxygenases studies have been mainly focused on higher plants. However, with more genomic data becoming available for other photosynthetic lineages (collectively referred to as algae), marine algae have a high potential to provide crucial insights into the evolution of life on the planet (Ulvskov et al., 2013). Our study gives first insights into LOX family evolution in brown algae. LOXs in brown algae have the closest relationship with prokaryotes, suggesting that extant LOXs in both groups derive from the same ancestral protein. They were divided into two clades. In each clade, Ectocarpales and Laminariales are distributed in different subclades, and LOXs in *S. japonica* have experienced several rounds of duplications. How new genes emerge and gain functionality is a long-standing fundamental question in biology. Gene duplication is a major mechanism for the evolution of novel genes and the development of phenotypic innovation (Lynch and Conery, 2000; Weissenbach et al., 2017). Our study shows that LOX duplication has occurred more frequently in *S. japonica* compared to Ectocarpales, forming 11 LOX genes. Proteome size-dependent scaling for the number of metal-binding proteins was observed across all of life, that is, the metal-binding proportion of a proteome, such as LOX, increases with the expansion of the proteome (Dupont et al., 2010). The predicted number of genes is 15,891 in *E. siliculosus*, 15,166 in *C. okamuranus*, and 18,733 in *S. japonica*, respectively. To some extent, the expansion of LOXs in *S. japonica* could therefore be related to the overall gene content expansion. Gene duplications can have one of three outcomes. One copy may simply be silenced (non-functionalization); one copy may acquire a new, beneficial function, with the other copy retaining the original function (neofunctionalization); or each copy may retain a subset of the original function, their total capacity being equal to the single ancestral gene (subfunctionalization) (Ohno, 1970; Lynch and Conery, 2000). Genes produced via gene duplication within a genome are called paralogs. The long-term retention of duplicated genes suggests either subfunctionalization of paralogous genes, or neofunctionalization (Lynch and Walsh, 2007; Weissenbach et al., 2017). Expression divergence is often the first step toward functional diversification. It reduces the chance of non-functionalization of duplicate genes and thereby increases the chance of duplicate genes being retained (Lan et al., 2009; Liu et al., 2015). Clear divergence in expression patterns was observed among brown algal LOXs in all three life stages. Even the most recently diverged paralogs (e.g., Ec-20_003610 *vs.* Ec-20_003620, SJ05859 *vs.* SJ12567, SJ16633 *vs.* SJ16634) differed greatly in their expression, suggesting that those duplicates have a relatively high rate of functional divergence. The *E. siliculosus* genome has a total of 879 duplicated gene pairs. Of these, there are 28 pairs where both members exhibited sex-biased expression in the same sex (Lipinska et al., 2015). Both pairs of *Ectocarpus* LOXs were all male-biased, providing a first indication for the potential roles of brown algal LOXs in development. Gene duplications are connected to genome evolution and in turn, morphological complexity (Rensing, 2014). Unlike *E. siliculosus*, which has an alternative life cycle with two isomorphic generations (both sporophyte and gametophyte are filamentous), *S. japonica* has two distinctive generations with microscopic gametophytes and large and complex sporophytes, which have initial tissue differentiation, such as holdfast, blade, and stipe (Kawai et al., 2016). *S. japonica* LOXs exhibit high divergence of expression in different life stages. They are most highly expressed in sporophytes, and four genes are only expressed in sporophytes. Li et al. (2016) demonstrated that 15-LOX activation promotes cell cycle progression via the generation of the lipid mediator 15-hydroxyeicosatetraenoic acid (15-HETE), which is important for fibroblast proliferation (Sutliff, 2017). 15-HETE also exists in kelps (Ritter et al., 2008). In *S. japonica* LOX genes are expanded and highly expressed in large sporophytes. This could potentially promote the rapid growth and the development of complex morphology via the formation of sufficient quantities of various lipid mediators.

Diversifying selection has been shown to drive expansions or contractions of gene families (Shiu et al., 2006; Gingerich et al., 2007; Lan et al., 2009). Genes involved in stress response, metabolism, and reproduction are frequently reported to be under positive selection, i.e., selection in favor of new genetic variants (Oliver et al., 2010; Koester et al., 2012). Positive selection promotes non-synonymous substitutions, with the ratio of non-synonymous (d*N*) to synonymous (d*S*) nucleotide substitutions d*N*/d*S* > 1. However, positive selection accounts for only a fraction of the retention of paralogs (Demuth and Hahn, 2009; Lan et al., 2009). Nucleotide substitutions are either under neutral (d*N*/d*S* = 1) or purifying selection (d*N*/d*S* < 1) if they are deleterious for a population (Teng et al., 2017). Purifying selection has predominated in the LOX family of brown algae, and we found no evidence even for episodic positive selection. Subfunctionalization may well explain why duplicate genes continue to be under purifying selection even after duplication. Selection against deleterious mutations in different functional domains of duplicate genes may promote the conservation of both duplicates and enhance the fixation of functional gene pairs (Lan et al., 2009; Tanaka et al., 2009). This may also apply to the brown algal LOX family. Moreover, LOX-mediated fatty acid transformations contribute to the formation of many brown algal pheromones and structural isomers, which might play a role in the chemical defense of these algae (Pohnert and Boland, 2002). As many as 23 different oxylipins have been found in the brown algal kelp *L. digitata* (Ritter et al., 2008). The more relaxed selection constraints in giant kelps, such as *S. japonica* might, to some extent, contribute to the formation of these diverse lipid products. Gene expression levels in yeast are inversely correlated to protein divergence. This relationship is hypothesized to exercise indirect control over mutation rates (Drummond et al., 2005). Positively selected genes are expressed at lower levels than genes subject to neutral or purifying selection and tend to be expressed in restricted conditions or specific tissues (Kosiol et al., 2008; Koester et al., 2012; Teng et al., 2017). Moreover, gene pairs with high d*N*/d*S* value tend to be more divergent in expression (Mock et al., 2017). Consistent with those studies, we found that LOX gene pairs with higher d*N*/d*S* values have lower expression levels than other LOX gene pairs both in *S. japonica* and *E. siliculosus*, though no positive selection was detected. The two pairs of genes with highest d*N*/d*S* values were the least expressed in all the three life stages of *S. japonica*. Three of these four genes were under relaxed purifying selection and exclusively expressed in sporophytes, while the fourth gene (SJ12567) has a higher expression level in male gametophytes. Lipinska et al. (2015) suggested that this sex-biased expression was promoted by an event where gene duplication has freed one member of the pair from selection pressure to evolve a sex-specific function. This gene is also the only one that was highly expressed in gametophytes, indicating a potentially important role in this life stage.

## Conclusion

Lipoxygenases constitute an important group of enzymes that metabolize PUFAs and produce various bioactive lipid mediators. LOXs in brown algae have closest relationships with those in prokaryotes. LOX gene expansion occurred in the macroalga *S. japonica*, mainly by tandem duplication and segmental duplication events. To investigate the evolutionary mechanisms responsible for the retention and subsequent functional divergence of duplicate LOX genes, we conducted molecular evolution, gene duplication, and expression analysis, and reconstructed the evolution history of this gene family in brown algae. LOXs in brown algae have undergone strong purifying selection. Expression of LOXs in *E. siliculosus* was up regulated under stress conditions. Expression also varied greatly between different LOX genes and life stages in brown algae, suggesting functional divergence since duplication. Our findings give new insights into the evolutionary history and functional diversification of brown algal LOX genes.

## Author Contributions

NY planned and designed the work. LT, WH, and XF analyzed and interpretated the data of the work. LT wrote the manuscript. DX, XZ, and SD revised it critically for important intellectual content. All authors approved the version to be published. All authors agree to be accountable for all aspects of the work in ensuring that questions related to the accuracy or integrity of any part of the work are appropriately investigated and resolved.

## Conflict of Interest Statement

The authors declare that the research was conducted in the absence of any commercial or financial relationships that could be construed as a potential conflict of interest.

## Footnotes

1 http://cctop.enzim.ttk.mta.hu/
