# Supplementary material for: Evolution and Expansion of the Prokaryote-Like Lipoxygenase Family in the Brown Alga Saccharina japonica
Source: Front Plant Sci. 2017 Nov 28;8:2018. doi: 10.3389/fpls.2017.02018 (PMC5712309; doi:10.3389/fpls.2017.02018)
Supplement: Supplementary file 2 [file Image_1.PDF]

Supplementary Material

Ancient lipoxygenase expanded in the brown alga *Saccharina japonica*

Linhong Teng[1], Wentao Han[1], Xiao Fan[1], Dong Xu[1], Xiaowen Zhang[1], Simon M. Dittami[2], Naihao Ye[1,3,]*

[1]Yellow Sea Fisheries Research Institute, Chinese Academy of Fishery Sciences, Qingdao, China, 266071

[2] Sorbonne Universit és, UPMC Universite ´Paris 6, CNRS, UMR 8227, Integrative Biology of Marine Models, Station Biologique de Roscoff, CS 90074, 29688 Roscoff Cedex, France

[3]Function Laboratory for Marine Fisheries Science and Food Production Processes, Qingdao National Laboratory for Marine Science and Technology, China, 266071

*Corresponding author: E-mail, yenh@ysfri.ac.cn; Tel/Fax: +86-532-85830360

1 Supplementary Figures

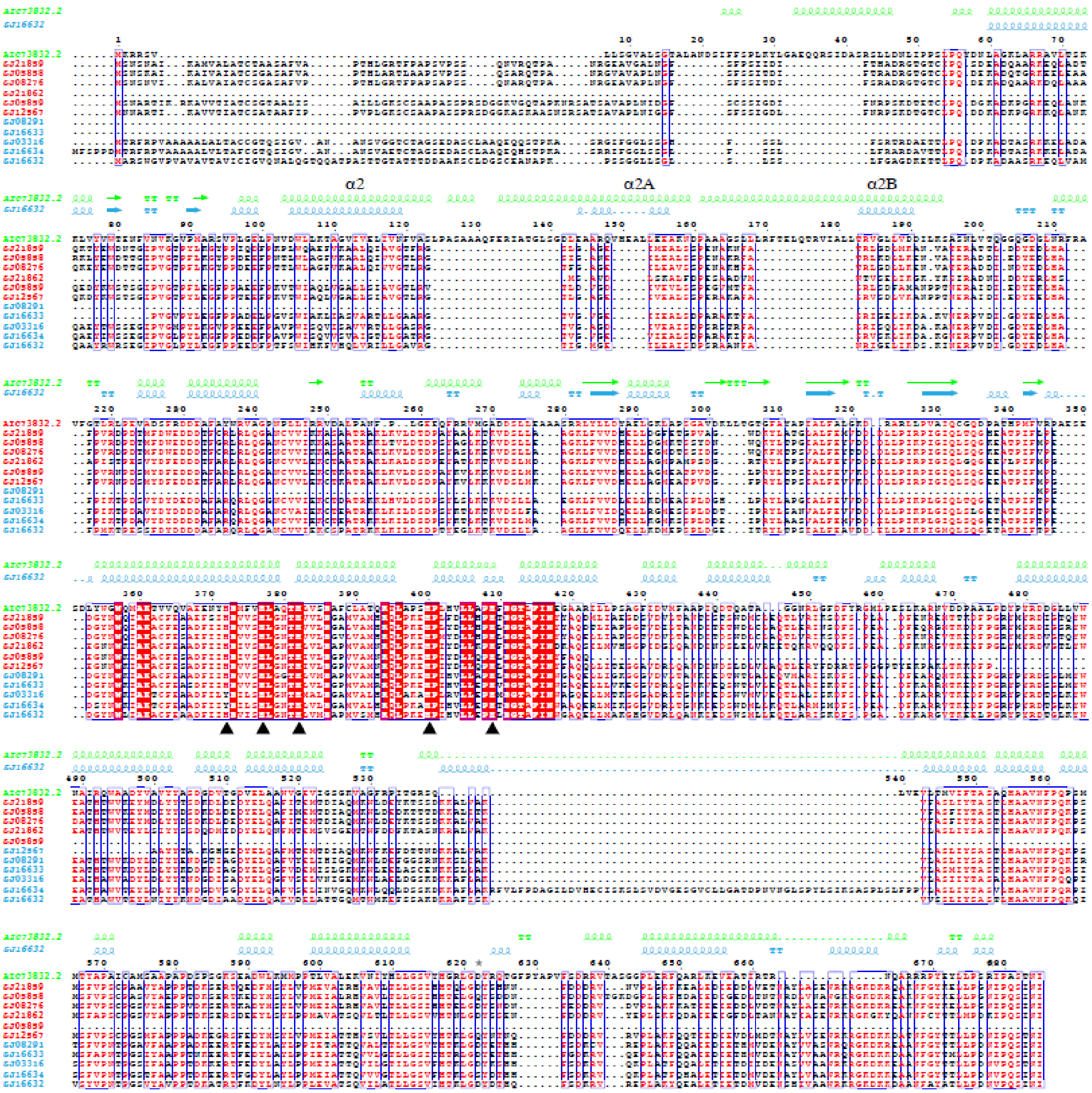

Supplementary Figure 1. The multiple sequence alignment of *S. japonica* and bacterial LOXs using the MUSCLE program (Edgar 2004). The graphical display of this alignment was generated, along with the added secondary structures above the alignment, using the ESPript web (Gouet, et al. 2003). The sequence names in green, red, and blue indicate that they are from *P. aeruginosa*, C1 and C2 in *S. japonica* respectively. The most conserved positions are shaded with red background, with black triangles at the bottom of colums indicating the conserved His motif. The secondary structures were extracted from the PDB structure of *P. aeruginosa* (PDB code 4g33) and from the predicted 3D structure of SjLOX (SJ16632). Long arrow: β-sheet; α: α-helix; TT: strict β turns.

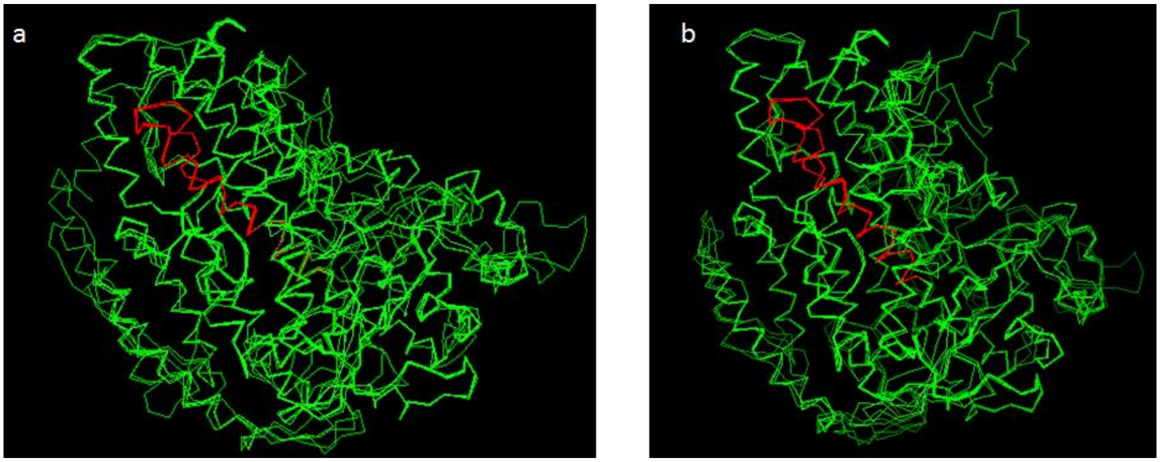

Supplementary Figure 2. Structural superposition of the six C1 LOX (**a**) and five C2 LOX (**b**). The red line represents the conserved HIS motif.
